# Diversification of myco-heterotrophic angiosperms: Evidence from Burmanniaceae

**DOI:** 10.1186/1471-2148-8-178

**Published:** 2008-06-23

**Authors:** Vincent Merckx, Lars W Chatrou, Benny Lemaire, Moses N Sainge, Suzy Huysmans, Erik F Smets

**Affiliations:** 1 Laboratory of Plant Systematics, K.U. Leuven, Kasteelpark Arenberg 31, P.O. Box 2437, BE-3001 Leuven, Belgium; 2 National Herbarium of the Netherlands, Wageningen University Branch, Generaal Foulkesweg 37, NL-6703 BL Wageningen, The Netherlands; 3 Centre for Tropical Forest Sciences (CTFS), University of Buea, Department of Plant & Animal Sciences, P.O. Box 63, Buea, Cameroon; 4 National Herbarium of the Netherlands, Leiden University Branch, P.O. Box 9514, NL-2300 RA, Leiden, The Netherlands

## Abstract

**Background:**

Myco-heterotrophy evolved independently several times during angiosperm evolution. Although many species of myco-heterotrophic plants are highly endemic and long-distance dispersal seems unlikely, some genera are widely dispersed and have pantropical distributions, often with large disjunctions. Traditionally this has been interpreted as evidence for an old age of these taxa. However, due to their scarcity and highly reduced plastid genomes our understanding about the evolutionary histories of the angiosperm myco-heterotrophic groups is poor.

**Results:**

We provide a hypothesis for the diversification of the myco-heterotrophic family Burmanniaceae. Phylogenetic inference, combined with biogeographical analyses, molecular divergence time estimates, and diversification analyses suggest that Burmanniaceae originated in West Gondwana and started to diversify during the Late Cretaceous. Diversification and migration of the species-rich pantropical genera *Burmannia *and *Gymnosiphon *display congruent patterns. Diversification began during the Eocene, when global temperatures peaked and tropical forests occurred at low latitudes. Simultaneous migration from the New to the Old World in *Burmannia *and *Gymnosiphon *occurred via boreotropical migration routes. Subsequent Oligocene cooling and breakup of boreotropical flora ended New-Old World migration and caused a gradual decrease in diversification rate in Burmanniaceae.

**Conclusion:**

Our results indicate that extant diversity and pantropical distribution of myco-heterotrophic Burmanniaceae is the result of diversification and boreotropical migration during the Eocene when tropical rain forest expanded dramatically.

## Background

Myco-heterotrophic plants present a number of unique challenges to those who are trying to understand their diversification and distribution. Many myco-heterotrophic plant species are rare and have very limited distribution ranges [1-4]. Their tiny, dust-like, seeds are assumed to be dispersed by wind or rainsplash [5]. This strategy seems ineffective for long-distance dispersal particularly because most species grow on the forest floor of dense primary rain forests. Furthermore their occurrence seems limited by their interaction with specific arbuscular mycorrhizal fungi, from which they obtain their organic carbon [6,7]. Paradoxally, some myco-heterotrophic genera are widely distributed often with remarkable disjunctions. Examples are *Sciaphila *(Triuridaceae), *Burmannia *and *Gymnosiphon *(Burmanniaceae), *Thismia *(Thismiaceae), *Voyria *(Gentianaceae) and *Monotropa *(Ericaceae), which all occur both in the New and the Old World [5]. While the disjunct distribution of *Voyria *has been interpreted as a result of a long-distance dispersal event [8], the widespread distributions of *Sciaphila*, *Burmannia*, *Gymnosiphon*, and *Thismia *were traditionally explained as an indication for a great antiquity, allowing vicariance explanation of the observed patterns [5,9,10]. This would imply that these genera originated before the breakup of western Gondwana, about 90–105 million years ago (Mya) [11,12]. A recent molecular dating analysis on monocots would not refute the western Gondwana vicariance hypothesis for Burmanniaceae (including Thismiaceae), as the stem and crown nodes of the family were estimated at 116 and 93 Mya respectively [13]. These dates would roughly put the relevant divergences in a Late Cretaceous timeframe, in particular when considering the generally large confidence intervals associated with molecular dating experiments in flowering plants [14]. The results of Janssen and Bremer [13], however, were based on 14 species of Dioscoreales, only three of which belong to the Burmanniaceae. Poor taxon sampling is one of the sources of error in molecular dating, though the effect of undersampling may depend on the method used to accommodate for rate variation [14-16].

The fossils used to calibrate the tree are another possible source of error in molecular dating [17]. Ambiguously interpretable morphology may result in the calibration of an erroneous node, and uncertain age of fossil-bearing rock may give rise to inaccurate dates [17,18]. Burmanniaceae are absent from the fossil record. A common approach in similar cases is to apply secondary calibration, i.e. use ages derived from other molecular dating estimates. This approach has been criticized for generating large confidence intervals [19]. We choose to rigorously expand our taxon sampling, including all monocot lineages, to estimate branch lengths based on one of the most widely available markers (18S rDNA), and to constrain the phylogeny to the most accurate phylogenetic hypotheses available in the literature. The date estimates of this single gene approach are compared with a Bayesian relaxed clock phylogenetic analysis [20] that uses secondary calibrations on a multi-gene Burmanniaceae dataset.

In this study we attempt to elucidate the diversification and biogeographic history of one of the most species-rich clades of myco-heterotrophic plants, the Burmanniaceae, by analyzing a thoroughly sampled data set containing nuclear and mitochondrial sequence data. The family consists of seven species-poor Neotropical genera and two species-rich genera with a pantropical distribution (*Burmannia *and *Gymnosiphon*). While the pantropical distributions of many angiosperm groups were traditionally interpreted as a result of tectonic vicariance, recent molecular dating studies have lead to a revival of long-distance dispersal theories [21,22]. Most recent studies on disjunct dispersal patterns between the Old and the New World of angiosperm families have strongly rejected vicariance as an explanation for the observed biogeographic pattern (e.g. Malpighiaceae [23], Rapateaceae and Bromeliaceae [24], Sapotaceae [25], Burseraceae [26], Melastomataceae [27], Moraceae 28], Meliaceae [18], *Renealmia *[29].

## Results

### Phylogenetic analyses

The three data partitions comprised the following numbers of taxa and characters: (1) 18S rDNA with 51 accessions, 1694 characters, and 222 parsimony-informative characters; (2) *nad1 b-c *intron with 49 accessions, 1645 characters, and 275 parsimony-informative characters plus Simple Indel Coding of the gaps adding 112 parsimony-informative characters; (3) ITS with 47 accessions, 598 characters, and 372 parsimony-informative characters.

**Figure 1:**
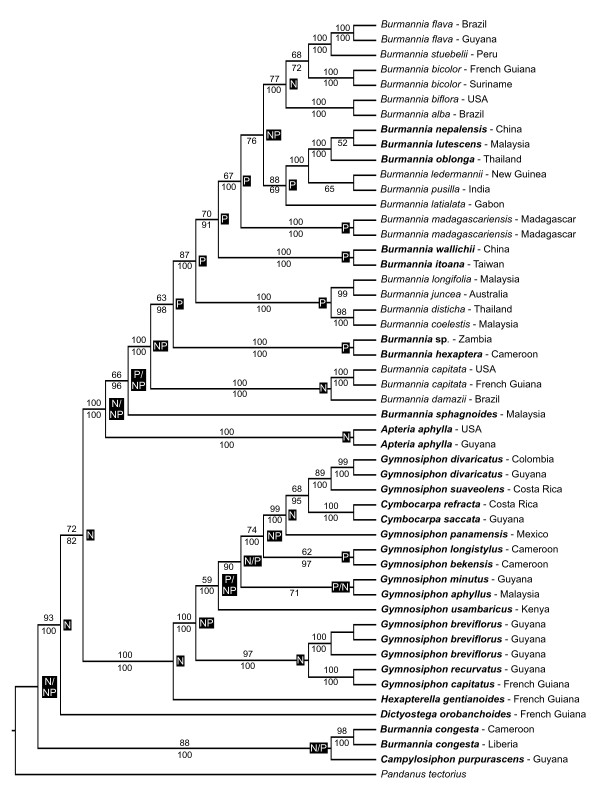
**Phylogenetic relationships of Burmanniaceae based on phylogenetic analysis of the 3-gene dataset**. One of the six most parsimonious trees for the combined 18S rDNA, ITS, and *nad1 b-c *data. Numbers above branches are bootstrap percentages from the maximum parsimony analysis. The Bayesian posterior probabilities are shown below branches. Black boxes show ancestral area reconstructions with DIVA. P and N indicate paleotropics and neotropics respectively. A slash separates two equally likely ancestral area hypotheses. Achlorophyllous species are shown in bold.

One of the six most parsimonious trees (tree length 4215; CI 0.522; RI 0.687) recovered during the parsimony analysis on the combined data is shown in Figure 1. No significant difference was observed between the maximum parsimony strict consensus tree and the Bayesian 50% majority-rule consensus tree. Most clades are well-supported (≥85% bootstrap support/≥95% Bayesian posterior probability). A well-supported clade with two samples of *Burmannia congesta *and *Campylosiphon *is sister to all other Burmanniaceae. Consequently, *Burmannia *is a paraphyletic genus. The neotropical genus *Dictyostega *is sister to the rest of the ingroup. *Apteria *is sister to the core *Burmannia *clade. Within the core *Burmannia *species *B. sphagnoides *is sister to the other species, consisting of two neotropical, two African, one Madagascan, and three East Asian clades. *Hexapterella *is sister to *Gymnosiphon*. *Cymbocarpa *is embedded in the *Gymnosiphon *clade. This clade consists of three neotropical, one East Asian, and two African lineages.

### Molecular dating

**Figure 2:**
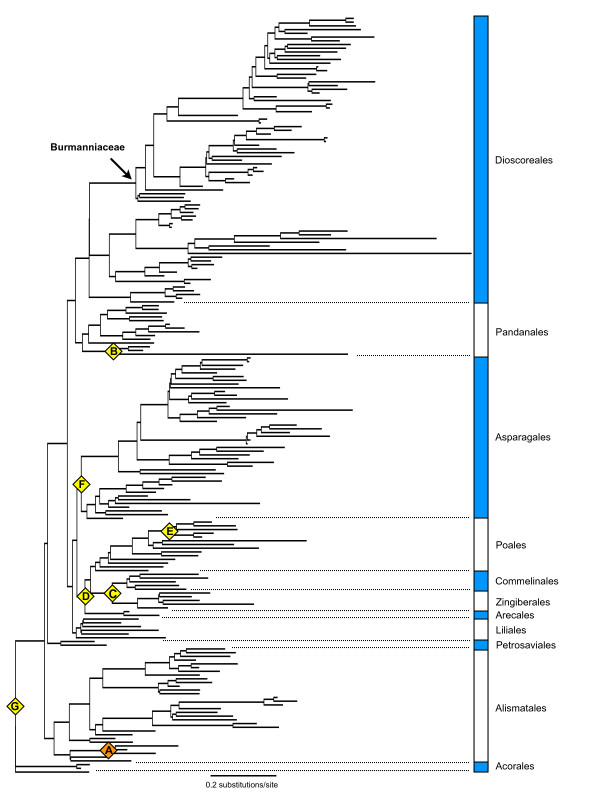
**Estimated branch lengths for 18S rDNA phylogenetic analysis of monocots**. Bayesian majority-rule consensus tree with optimized branch lengths based on 18S rDNA sequences of 202 monocot taxa and *Amborella *as outgroup. This tree was used as input for the divergence time estimations with penalized likelihood. The considered calibration points (A-G; see text) are plotted on the tree.

**Figure 3:**
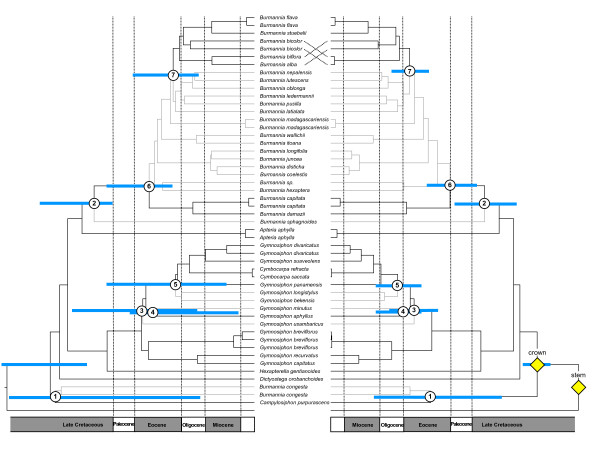
**Divergence time estimates of Burmanniaceae**. Evolutionary chronograms of Burmanniaceae. The left-side tree is derived from the 18S rDNA Bayesian tree of sampled monocots and the penalized likelihood relaxed molecular clock analysis. From this tree covering all monocot lineages, only the Burmanniaceae clade is shown here. The right-side tree results from the multi-gene BEAST relaxed clock analysis with secondary calibration points. The nodes that were constrained (secondary calibration points) are indicated with yellow squares. Paleotropical lineages are indicated with grey branches, black branches denote neotropical lineages. The nodes labelled 1–7 correspond to the origin of New/Old World disjunctions.

The 50% majority-rule consensus tree of the constrained Bayesian analysis of the 18S rDNA monocot dataset with optimized branch lengths is shown in Figure 2. The age estimations with their standard deviations and credibility intervals obtained for the monocot orders Acorales, Alismatales, Petrosaviales, Dioscoreales, Pandanales, Liliales, Asparagales, Arecales, Zingiberales, Commelinales, and Poales are listed in Table 1. The stem-node age of the Burmanniaceae is estimated to 116 ± 2.6 Mya, the crown node to 96 ± 3.37 Mya. The Burmanniaceae clade clipped from the r8s chronogram of the monocots is shown in Figure 3. The crown and stem age estimates of the Burmanniaceae were used as secondary calibration points for the multigene analysis with BEAST. The chronogram resulting from this analysis is also shown in Figure 3.

**Table 1:** Age estimation of monocot orders.

Order		**Penalized Likelihood age estimations (in Mya)**	Credibility intervals	**Janssen and Bremer (2004) age estimations (in Mya)**^1^
Acorales	*stem node*	134^2^	134^2^	
	*crown node*	19 ± 5.7	7 – 44	
Alismatales	*stem node*	128 ± 1.7	123 – 133	131
	*crown node*	123 ± 3.9	97 – 133	128
Petrosaviales	*stem node*	128 ± 1.9	121 – 132	126
	*crown node*	108 ± 7.11	87 – 102	123
Dioscoreales	*stem node*	121 ± 2.1	119 – 130	124
	*crown node*	116 ± 2.6	113 – 126	123
Pandanales	*stem node*	121 ± 2.1	119 – 130	124
	*crown node*	117 ± 2.4	116 – 130	114
Liliales	*stem node*	122 ± 2.6	109 – 131	124
	*crown node*	118 ± 6.03	78 – 131	117
Asparagales	*stem node*	122 ± 4.7	98 – 126	122
	*crown node*	119 ± 4.1^4^	101 – 127	119
Arecales	*stem node*	116 ± 5.1^5^	94 – 122^3^	120
	*crown node*	51 ± 14.6^6^	15 – 98	110
Commelinales	*stem node*	92 ± 6.8	83 – 114	114
	*crown node*	75 ± 8.8	50 – 104	110
Zingiberales	*stem node*	92 ± 5.5	91 – 116	114
	*crown node*	67 ± 7.1	52 – 96	88
Poales	*stem node*	109 ± 5.2	89 – 120	117
	*crown node*	106 ± 5.3	88 – 116	113

Age estimations of monocot orders obtained with PL analysis of 18S rDNA data, including credibility intervals. The age estimations by Janssen and Bremer [13] are listed for comparison.

^1 ^The analysis of Janssen and Bremer [13] is based on 878 *rbcL *sequences. Dating was obtained with nonparametric rate smoothing (NPRS).

^2 ^This node was fixed at 134 Mya.

^3 ^Only one *Acorus *accession was used in the analysis of Janssen and Bremer [13].

^4 ^This node was constrained to a minimum age of 93 Mya.

^5 ^This node was constrained to a minimum age of 89.5 Mya.

^6 ^Only two Arecales accessions were included in the analysis.

### Tempo of diversification

**Figure 4:**
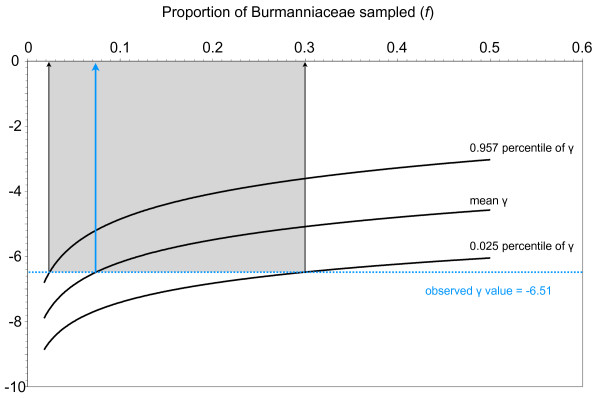
**The relationship between γ and the proportion of extant taxa in Burmanniaceae (*f*)**. We sampled 41 species of Burmanniaceae and measured a γ-value of -6.51 using the BEAST chronogram (blue dotted line). To explore the effects of missing lineages we generated γ-statistic values under the assumption that our sampling n (n = 41) represents only a proportion (*f*) of the extant Burmanniaceae species. The three curves show the mean, 0.975 percentile, and 0.025 percentile of the γ-distribution. This distribution was constructed by calculating γ of 1000 simulated pure-birth phylogenies with n/*f *tips, each randomly pruned to 41 tips, for values of *f *ranging from 0.018 to 0.5. To measure a γ-value of -6.51 using 41 taxa while the actual value is not significantly different from zero, Burmanniaceae should contain 562 lineages (95% CI: 139–1708). Currently 92 Burmanniaceae species are known [60].

We measured a negative γ value for our chronogram (γ = -6.51), which rejects the hypothesis that rates of lineage accumulation in Burmanniaceae remained constant over time, in favour of a decrease of speciation rate through time [30]. Simulations indicate that with a sample of 41 species for the γ-statistic to yield – 6.51 when the true value is zero, there would need to be 562 species of Burmanniaceae (95% CI: 139–1708) (Figure 4). We can therefore reject the possibility that the negative γ-value is the result of a poor sampling artefact.

### Lineage-through-time plot

**Figure 5:**
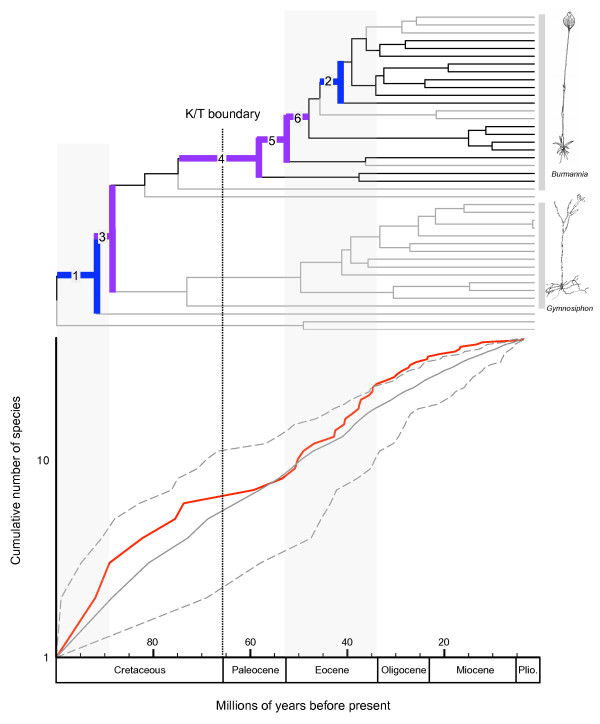
**Estimated timing and tempo of diversification in Burmanniaceae**. (a) BEAST chronogram of the ingroup with multiple accessions for the same species excluded. Grey branches indicate achlorophyllous species. Branches with significant diversification rate shifts are numbered; shifts 1 and 2 are supported by Δ1, Δ2, and Slowinski and Guyer statistics; shifts 3–6 are supported by the relative cladogenesis statistic. *Burmannia *(excluding *B. congesta*) and *Gymnosiphon *clades are highlighted with grey bars, with two drawings of specimens as exemplars. (b) Semilogarithmic lineage-through-time (LTT) plot of Burmanniaceae (red) and simulated LTT plot with 95% confidence intervals (grey) with a constant death-birth rate of 0.5. Upturns or downturns in the empirical LTT plot reflect changes in diversification rate. Prolonged periods of increased diversification rate are highlighted by shaded areas.

A semilogarithmic lineage-through-time (LTT) plot (Figure 5) shows a trend toward reduced diversification rates beginning ≈72 Mya (Late Cretaceous), and an Eocene increase of diversification rate. A model of gradual change in diversification rate (model B, γ = 1.65, AIC = 360.15) was chosen over model A (AIC = 371.05) and model C (AIC = 391.02) as best fit of the empirical LTT plot using AIC. Model B was preferred no matter which timing of an abrupt rate shift in model C was specified. The hLRT showed significant difference between model A and B (P = 0.0004), while there was no significant difference between model A and C.

Significant diversification rate shifts were detected in two branches by the Δ1, Δ2, and the Slowinski and Guyer (SG) statistic (Figure 5, branches 1 and 2). The relative cladogenesis (RC) test identified four different branches with rate shifts (Figure 5, branches 3–6). The shifts identified by RC should be treated with caution as the RC test is sensitive to temporal depth, phylogenetic scope, and the non-independence of diversification rate shifts [31].

### Analysis of ancestral areas

The optimal solutions obtained with the DIVA analysis calculated three equally likely possibilities for the distribution of the most recent common ancestor of the Burmanniaceae: 1) the Amazonian Region; 2) the Guineo-Congolean and Amazonian Region; 3) the Amazonian and Brazilian Region. Thus despite the lack of resolution to precisely identify the ancestral area of Burmanniaceae, the analysis indicates it was western Gondwanan. According to the second DIVA analysis seven New-Old World dispersals are required to explain the distribution of the terminals. But again, the ancestral area of Burmanniaceae cannot be tracked solely to the paleotropics or neotropics (Figure 1). We assigned seven nodes that may represent dispersal or vicariance events between the neotropics and the paleotropics (Figure 3). Due to the ambiguous optimization of the ancestral areas events 2 and 3 could equally likely be placed on adjacent deeper nodes yet this would still place these events in the same geological epoch.

## Discussion

### Divergence time estimates

The araceous fossil *Mayoa portugallica *[32] was identified as the most inconsistent calibration point relative to the other calibration points and was therefore not used in the PL analysis, which estimated the node at 64 Mya or 42% younger than suggested by fossil data. This observation does not necessary imply that the fossil was assigned to a wrong node. There are many possible explanations for this result (for example, error in branch length estimation, failure of the rate smoothing method to comply with the 18S rDNA rate heterogeneity, underestimation of the true age by the other fossils as a result of an incomplete fossil record) [17,33]. A rerun of the PL analysis revealed that calibration point A had hardly any impact on the age estimations of the Burmanniaceae. With calibration point A included Burmanniaceae age estimations were overall less than 1% older (results not shown). Apparently the long phylogenetic distance of calibration point A from the Burmanniaceae clade or the influence of calibration points in more closely related clades reduces the impact of this calibration point.

The obtained divergence time estimations of the monocot orders, using a different marker (18S rDNA instead of *rbcL*) and considerably fewer taxa, are mostly younger than those published by Janssen and Bremer ([13] see also Table 1). For most orders these differences in age estimations are small. However, the estimations of both crown and stem nodes of some more derived orders (Arecales, Commelinales, and Zingiberales) are considerably younger than those of Janssen and Bremer [13] (Table 1). We attempted to sample the earliest-diverging lineages of each order as well as some more derived taxa. But the much lower taxon sampling in our study compared to the more than 800 terminals in Janssen and Bremer [13] is likely to have caused an overestimation of the branch that subtends the indicated clades (or underestimation of the branches within these clades) and as a result has led to underestimation of the ages of the crown nodes [15,16]. The stem and crown node ages of Dioscoreales are consistent with a recent estimate that used a similar calibration strategy on a two-gene dataset (18S rDNA and *atpA*) but with fewer taxa [34].

In general the use of secondary calibration points for divergence time estimates is not favored [35]. However, this strategy becomes more sound when the error associated with molecular dating estimates can be transferred into the subsequent divergence time analysis. Here this is coupled with the advantage of accommodating unlinked rate variation across all available loci (a 'multigene' approach). Not unexpectedly, the BEAST analysis results in a chronogram with slightly less branch length heterogeneity than the PL chronogram. 18S rDNA sequences alone do not support a fully resolved Burmanniaceae clade. Artificially resolved clades (by constraints) will result in short branch lengths and more compressed divergence patterns if the data provides insufficient phylogenetic signal to support these clades. This effect seems limited here: the age estimations of the BEAST analysis are not significantly different from those inferred by PL (Figure 3). Node 1 encompasses the most pronounced difference between the two strategies. While PL assigns this node to the Cretaceous, BEAST favors an Eocene origin, but the credibility intervals on both estimates are large and overlapping.

### Biogeography and diversification of Burmanniaceae

This study points towards a West Gondwanan origin of Burmanniaceae. According to our results the family started to diversify 96.4 Mya (mid-Cretaceous), well before the K/T boundary. The diversification rate shift tests and the LTT plot suggest a high initial diversification rate. Mid-Cretaceous climate was relatively warm [36] although fossil data suggests tropical forests were open and dry adapted and modern closed-canopy rain forest did not originate until after the K/T boundary [37]. However, recent molecular studies suggest that closed-canopy rain forest existed during the mid-Cretaceous [38]. The most recent common ancestor of Burmanniaceae was photosynthetic (see further) and it is possible that initial diversification of the family started when Burmanniaceae lineages became parasitic on arbuscular mycorrhizal fungi as an adaptation to shaded habitats [39]. The separation between South America and Africa has been dated to 105 Mya [12], but the biogeographical timing of this continental breakup is somewhat uncertain because it is unclear when an effective dispersal barrier was established [40]. Furthermore, stepping-stone dispersal routes between South America and Africa may have delayed biogeographical isolation into the Late Cretaceous [41]. Because the *Burmannia congesta-Campylosiphon *clade is the earliest-diverging lineage in Burmanniaceae one proposed ancestral area of the family includes both neotropics and paleotropics (Figure 1). Under this assumption the separation between Africa and South America is likely to be reflected in the divergence time hypotheses. While such a vicariance event is indeed suggested by the direct dating approach with PL (node 1 = 94 Mya), BEAST analysis favours an Eocene divergence (node 1 = 47 Mya). Due to the large error associated with both estimates none of the hypotheses can be rejected. A Cretaceous continental drift scenario thus could explain the split between *B. congesta *and *Campylosiphon*, however, such a vicariance event has been observed only rarely in flowering plants [42-44]. Contrasting this hypothesis, an Eocene boreotropical migration scenario (see further) would be an equally possible explanation for the disjunction observed in node 1. The DIVA analysis supports a neotropical origin for the clade containing all other Burmanniaceae lineages. The Malaysian species, *Burmannia sphagnoides *branched off from its neotropical predecessors 75 Mya (PL) – 72 Mya (BEAST) ago (Figure 3, node 2). Migration between Gondwana and Laurasia in the Late Cretaceous could have been possible through land connections allowing the interchange of taxa between South America, India, and Madagascar [12,45,46]. Furthermore, India may have acted as a raft that transported some taxa from Madagascar to Asia [47]. Both phenomena together could well explain the biogeographical history of *B. sphagnoides*.

The Late Cretaceous and Paleocene are characterized by a decrease in diversification rates (Figure 5). This slowdown may have resulted from decreased origination rates (e.g. filling ecological niches, [48]), and/or from increased extinction rates. The latter hypothesis is coincident with the occurrence of the K/T mass-extinction [49,50]. A subsequent increase of the diversification rate, associated with the diversification *Burmannia *and *Gymnosiphon*, occurred at the Late Paleocene and through the Eocene. Simultaneously lineages of both genera reached the Old World (Figure 3). These events are well correlated with a boreotropical dispersal scenario as proposed for other tropical angiosperm families [18,23,25-28]. According to this scenario South American taxa of *Burmannia *and *Gymnosiphon *are hypothesized to have migrated via scattered, continental and/or volcanic islands that connected North and South America at various times during the Tertiary [23]. From North America migration across Laurasia in to the Old World could continue via a series of connections across the North Atlantic ('North Atlantic Land Bridge' [23,51]. Further diversification into Africa, Madagascar, and Asia would explain the current distribution patterns of these genera. Laurasian migration of tropical groups was facilitated by warm and humid climates that occurred during the Eocene [52]. Particularly at the beginning of the Eocene global temperatures peaked and plants with tropical affinities grew at middle and high latitudes [53,54]. This is also the reason why an eastern migration through Beringia, which did not support tropical vegetation, seems unlikely [23,26]. Significant cooling during the Oligocene caused a retraction of the boreotropical flora from across the North Atlantic [55]. This cooling, which started at the end of the Eocene [51], probably caused a 'climatic' vicariance in *Burmannia *and *Gymnosiphon*: as both distributions moved southwards they became separated into New and Old World groups (Figure 3, nodes 5 and 7). According to our results, no migration between the neotropics and paleotropics occurred for the remaining ≈30 My, illustrating the limited long-distance dispersal capabilities of Burmanniaceae species. Simultaneously with the Oligocene cooling the LTT plot shows the start of a gradual decrease of the diversification rate towards the present. An overall decrease in lineage accumulation for Burmanniaceae is also suggested by the CR test.

### Loss of chlorophyll

One of the most intriguing features of Burmanniaceae is the absence of chlorophyll in most taxa, except for some *Burmannia *species. Almost nothing is known about the chloroplast genome in achlorophyllous Burmanniaceae or myco-heterotrophic plants in general. Studies on the chloroplast genome of parasitic plants reported the loss of most chloroplast genes [56,57]. If we assume that the chloroplast genes in myco-heterotrophic Burmanniaceae undergo the same fate, then a reversal to autotrophy seems highly improbable. With this assumption, at least eight independent losses of chlorophyll took place in Burmanniaceae (Figure 5). As each lineage may have lost its chlorophyll independently, it is difficult to speculate about the age of these events. According to a most-parsimonious pattern, the ancestral lineage leading to the *Gymnosiphon-Hexapterella *clade lost its photosynthesis during the Late Cretaceous. The evolution from a mycorrhizal photosynthetic plant towards a non-photosynthetic myco-heterotroph has been explained as a phenomenon that can provide escape from competitive exclusion in the shaded conditions of forest understory habitats [39]. A Late Cretaceous origin of myco-heterotrophy thus provides evidence for the presence of closed-canopy environments before the K/T boundary [38]. The chlorophyll losses in the core *Burmannia *clade would have occurred during the Eocene and the Oligocene, when closed-canopy rain forest was abundant even at low latitudes [53,58]. While *Burmannia *species seem to have lost their chlorophyll after crossing the North Atlantic Land Bridge, a single loss event in the *Gymnosiphon *clade would have occurred before their Laurasian migration. This result suggests that achlorophyllous plant species were able to migrate and diversify long after their adaptation to a myco-heterotrophic nutrition strategy.

## Conclusion

In his excellent monograph of the Burmanniaceae Jonker [10] wrote: "Fossil Burmanniaceae are unknown. The family however is very old, according to the occurrence of closely related species in America, Africa, and Asia [...]". Our analyses reveal that Burmanniaceae are a relatively old family, and vicariance events possibly influenced the early diversification of the family. Our study also suggests that the diversification and radiation of the pantropical genera *Burmannia *and *Gymnosiphon *started from South America during the Eocene when continental drift had separated South America from Africa. The global temperature during that epoch was high enough to allow tropical rain forests to expand significantly. This triggered an increased diversification in *Burmannia *and *Gymnosiphon *and allowed for boreotropical migration across the North Atlantic Ocean in to the Old World for both genera. Our results imply that the increase of neotropical plant diversity during the Eocene [58] and the boreotropical migration of tropical plants [23-29] also applies to myco-heterotrophic plants.

## Methods

### Molecular data

This study samples 41 species of Burmanniaceae, covering seven of the nine genera [10,59,60]. Only the monospecific neotropical genera *Marthella *and *Miersiella *are not represented. For *Burmannia *our sampling includes 23 of the approximately 60 described species (38%), for *Gymnosiphon *12 species of 24 known species were available (50%) [60]. With species from the New World, Africa, and Asia our sampling covers the current geographic distribution range of both genera. For the phylogenetic inference of Burmanniaceae, sequence data of *Pandanus tectorius *(Pandanales) were used as outgroup. Herbarium vouchers and GenBank accessions for the taxa used in this study are listed in Additional file 1.

Sequence data of 18S rDNA and *nad1 b-c *from a previous study [61] were supplemented with additional sequences. For most species ITS data was obtained using the following protocol. DNA was extracted from silica dried and herbarium material with the PureGene DNA extraction kit (Gentra Systems, Landgraaf, The Netherlands) following the manufacturer's instructions. The nuclear 18S rDNA region and the mitochondrial *nad1 b-c *intron were amplified following Merckx et al. [61]. Amplification of the nuclear ITS region was carried out with the primers ITS1 and ITS4 [62], with a premelt of 5 min at 94°C, followed by 30 cycles of 30 s of denaturation at 94°C, 30 s annealing at 55°C, 1 min extension at 72°C, and a 7 min final extension at 72°C. All PCR products were cleaned with the Nucleospin Extract II columns (Machery-Nagel, Düren, Germany) following manufacturer's instructions. Sequencing reactions were run on an ABI 310 automated sequencer (Applied Biosystems, Fostercity, USA). Some samples were sequenced by the Macrogen sequencing facilities (Macrogen, Seoul, South Korea). Sequencing files were edited and assembled using Staden for Mac OS X [63].

Due to contamination with fungal DNA no ITS data could be obtained for three taxa: *Burmannia sphagnoides*, *Gymnosiphon suaveolens*, and *G. panamensis*. In the *nad1 b-c *dataset sequences of *B. nepalensis *and *C. saccata *are missing. The *nad1 b-c *sequence of *C. saccata *(DQ786096) was not used as this sequence probably belongs to a *Gymnosiphon *species.

Alignment was done by eye using MacClade 4.04 [64]. Gaps in the *nad1 b-c *intron data were coded using the simple indel coding method (SIC; [65]) as implemented in SeqState [66]. Autapomorphic indel characters were manually removed from the dataset.

### Phylogenetic analyses

Molecular data were analyzed using maximum parsimony and Bayesian methods. Each of the three data partitions (18S rDNA, ITS, and nad1 b-c [including indel characters]) was analyzed separately. Since no strongly supported (>85% bootstrap percentage/>95% Bayesian posterior probability) incongruences were observed between the topologies, combined analyses of the molecular data were performed. Maximum parsimony (MP) analyses were done with PAUP* v4b10 [67] using a heuristic search with the TBR branch swapping algorithm for 1,000 replicates, holding 5 trees at each step and with the Multrees option in effect. Branch stability was calculated using a bootstrap analysis with 1,000 pseudo-replicates. For each replicate a heuristic search was conducted with the same settings as described above. Model selection for the Bayesian analyses was done using Modeltest v3.06 [68]. For all tree genes Modeltest selected the GTR+I+G model. For the indel data we selected the restriction site model as recommended in the MrBayes 3.1 manual [69]. The combined analyses were performed with a partitioned model approach. Bayesian analyses were run on the K.U. Leuven UNIX cluster ('VIC') using MrBayes 3.1.2 [70,71]. Each analysis was run three times for three million generations sampling every 1,000 generations. The first 50% of the sampled trees were treated as burnin and discarded. The sump command in MrBayes was used to check whether the two separate analyses converged on similar log-likelihoods. Additionally convergence of the chains was checked using TRACER 1.4 [72] and the effective sampling size (ESS) parameter was found to exceed 100, which suggests acceptable mixing and sufficient sampling.

### Divergence time estimation

**Figure 6:**
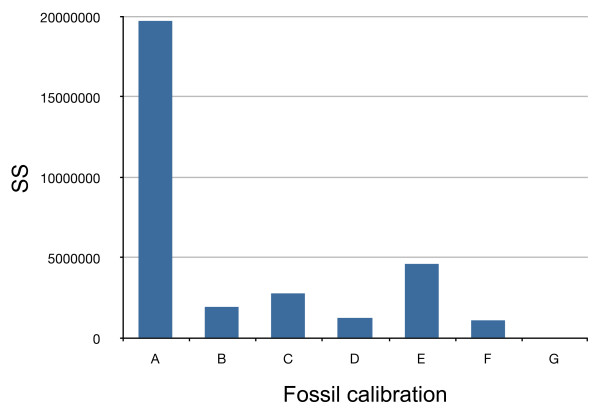
**Consistency of calibration points**. Histogram of the summed square values of the deviations between molecular and fossil ages (SS) for each calibration point.

**Figure 7:**
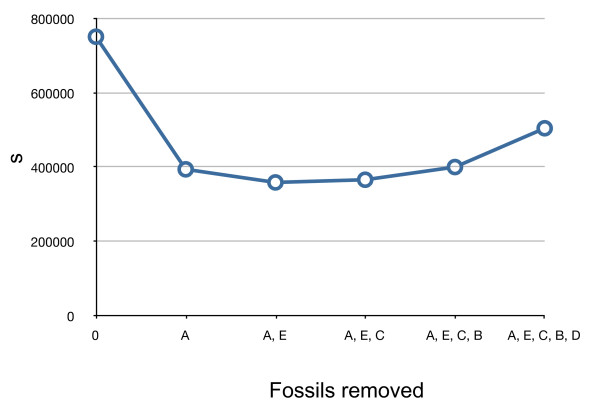
**Effect of calibration point removal**. Plot illustrating the effect of removing fossil calibration points on the average squared deviation (s).

Burmanniaceae and Dioscoreales in general are absent from the fossil record [73] and because our sampling does not allow dating based on geographic history (e.g. volcanic islands), it is impossible to calibrate the Burmanniaceae tree directly. To estimate ages of nodes in the Burmanniaceae phylogeny we expanded our phylogeny to comprise all monocot lineages. This allowed the incorporation of multiple fossil calibration points, in order to minimize bias produced by single calibration points. An additional purpose was to have fossils calibrating nodes at different distances to the root of the phylogeny, averaging out any biases that might result from calibrating at different levels in the phylogeny. To this end we extended our 18S rDNA sampling with 18S rDNA accessions from GenBank of all monocot orders and *Amborella *as outgroup [see Additional file 1]. This dataset of 203 taxa and 1662 characters was analyzed with MrBayes using the following constraints: (1) all monocot orders were forced to be monophyletic; (2) the relationships between the orders was constrained according to the multi-gene monocot topology by Chase [74], and (3) the relationships between the Burmanniaceae taxa were constrained to the multi-gene tree presented in this study. The Bayesian analysis was run for five million generations, sampling every 1,000 generations, and using the GTR+I+G model as selected by the AIC implemented in Modeltest 3.06. A majority-rule consensus tree with branch lengths averaged over the last 2,500 trees (50%) was obtained with the sumt command. Branch lengths of this majority-rule consensus tree were then optimized with MrBayes under the GTR+I+G model by setting the proposal probability ('props') of the 'node slider' to 5 and the proposal probability of all other topology moves to 0 [69]. Because MrBayes requires a fully resolved starting tree polytomies in the majority-rule consensus tree were arbitrarily resolved. The MCMC was run over two million generations, sampling every 1,000 generations. A majority rule tree was calculated over the last 1,000 sampled trees (Figure 1). Because a χ^2 ^likelihood ratio test strongly rejected a strict molecular clock for our data (χ^2 ^= 1316.36; df = 201; P = 6.6 × 10^-150^), we applied a relaxed clock model, using penalized likelihood (PL) analysis as implemented in r8s [75] to obtain age estimations. Seven calibration points were used to calibrate the 18S rDNA tree (Figure 2). A: a minimum age constraint of the stem node of Monsteroideae (Araceae) of 110 Mya. This is consistent to the minimum age of *Mayoa portugallica*, an araceous fossil assigned to the tribe Spathiphylleae [32]. B: a minimum age constraint of 90 Mya to the crown node of Triuridaceae. This is based on the Triuridaceae fossil flowers *Mabelia *and *Nuhliantha *from the Upper Cretaceous [76]. Phylogenetic analyses of morphological data showed that both fossil genera are nested within extant Triuridaceae. Our sampling includes two species of *Sciaphila *and one *Kupea *species. The latter genus was shown to be the basally-divergent node of Triuridaceae [77]. C: the minimum age of the split between Zingiberales and Commelinales constrained to 83 Mya [78,79]. D: the stem node age of the Arecaceae constrained to a minimum of 89.5 Mya [79,80]. E: the crown node of the Flagellariaceae/Poaceae/Joinvilleaceae/Restionaceae clade constrained to a minimum age of 69.5 Mya [79,81]. F: a minimum age of 93 Mya for the Asparagales crown node based on the minimum age of fossil *Liliacidites *pollen [82]. G: all calibration points listed above are fossil data and therefore apply minimum ages only. However, the r8s software requires at least one fixed calibration point. In order to achieve this we considered a fixed crown node age of the monocots of 134 Mya, an estimate obtained by Bremer [79]. This age is consistent with results obtained by other studies [83-85]. We used the 'fossil cross-validation' method to measure the agreement between these different calibration points [17]. This method compares the difference between the fossil and molecular ages by rerunning the PL analysis with single calibration nodes. The summed square (SS) values of the deviations between molecular age estimations and the fossil constraint's age for each node are plotted in Figure 6. Calibration point A exhibited the largest SS. Removal of this calibration point resulted in a two-fold decrease of the average squared deviation (*s*) of all remaining fossils (Figure 7). Subsequent removal of the other fossil calibrations had no impact on the magnitude of *s*. Fossil constraint A was therefore identified as the most inconsistent calibration point and omitted from the analyses.

The rooted Bayesian majority-rule consensus tree with optimized branch lengths was used as the input tree for a penalized likelihood (PL) analysis with r8s 1.70 [75,86]. As suggested by the author, the truncated-Newton (TN) optimization algorithm was selected for the analyses. The rate smoothing penalty parameter was set to 1.3e^2 ^as determined by the statistical cross-validation method implemented in r8s 1.70. Standard deviations and credibility intervals on the age estimations were calculated by reapplying PL to 1,000 randomly chosen trees kept from the Bayesian analysis.

Additionally we performed a Bayesian relaxed clock analysis with all three data partitions using BEAST 1.4.6 [87]. We applied a GTR+I+G model with 4 gamma categories on each partition following the 'BEAST partitioning manual' [88]. The uncorrelated lognormal clock model [20] was selected and two secondary calibration points and the credibility intervals were taken from the r8s analysis described above: a prior of 116.3 ± 2.6 Mya was set for the root of the tree and a prior of 96.0 ± 3.4 Mya for the crown node of the Burmanniaceae. In the BEAST analysis a normal distribution was applied to reflect the credibility intervals produced by reapplying PL to 1,000 trees. The distribution of all other priors was set to uniform. Posterior distributions of parameters were approximated using two independent Markov chain Monte Carlo analyses of twenty million generations followed by a discarded burnin of 2,000,000 generations (10%). Convergence of the chains was checked using TRACER 1.4 [72] and the effective sampling size (ESS) parameter was found to exceed 100, which suggests acceptable mixing and sufficient sampling. The XML BEAST input file is available from the first author on request.

### Tempo of diversification

We evaluated the tempo of lineage accumulation in Burmanniaceae using the constant-rate (CR) test [30]. This test uses the γ-statistic to compare the relative positions of the nodes in the chronogram to those expected under a CR model of diversification. A negative value of the γ-statistic indicates that that nodes are closer to the root than expected under a CR model and implies a deceleration in the accumulation of lineages. A positive value indicates that nodes are closer to the tips than expected under a CR model and implies an acceleration of lineages [30]. A CR model of diversification can be rejected at the 95% level if γ < -1.645 [30]. We calculated the gamma-statistic of the BEAST chronogram of the ingroup with duplicate species excluded. However, the γ-statistic is biased by extinction, because older lineages have higher risks of being extinct at present than younger ones (bias towards positive γ values), and by incomplete taxon sampling, because nodes near the root of the tree give rise to more extant descendants than nodes near the tips and are therefore more likely to be included in a small random sample (bias towards negative γ values) [89,90]. To explore the effects of incomplete taxon sampling on the γ-statistic calculated on the Burmanniaceae chronogram we simulated pure-birth (d:b = 0) trees with Phylogen 1.1 [91] under the assumption that our Burmanniaceae sampling n (n = 41) represents only a proportion (*f*) of the actual diversity [92,93]. 1,000 pure-birth trees were simulated with n/*f *tips for *f *values ranging from 0.018 to 0.5. This corresponds to an extant Burmanniaceae diversity ranging from 82 to 2278 species. Each tree was then randomly pruned to 41 tips and the γ-statistic was calculated. For each simulated dataset of 1000 phylogenies the mean value of γ and the 95% confidence interval were plotted to estimate the 95% confidence interval for the number of lineages that must be missing to obtain a γ-statistic as extreme as the measured one if γ actually is zero [93]. The resulting curves are shown in Figure 4. All γ-statistics were calculated with Genie 3.0 [94].

### Lineage-through-time plot

A lineage-through-time (LTT) plot of the BEAST chronogram without doublet species was constructed with END-EPI [95]. Our sampling consisted of ≈45% of described Burmanniaceae lineages (41 out of 92 species [60]). To evaluate the effects of incomplete taxon sampling on the slope of the LTT plot, we generated 1,000 phylogenies with 92 taxa under a death-birth ratio of 0.5, and randomly pruned each tree to 41 taxa. The branch lengths of the resulting trees were scaled with TreeEdit 1.0 [95] to set the root node of each tree 96.4 My from the tips (the crown node age of the Burmanniaceae estimated using BEAST). The scaled trees were used to construct a mean LTT curve with 95% confidence intervals. To evaluate the fit of the empirical LTT plot to three general models of diversification (A, B, and C [97,98]) we used the different survival models implemented in the APE 1.8 package [97,99]. Model A assumes a constant diversification rate; Model B assumes a monotonically changing diversification rate. The parameter that controls the change of this rate is called γ. If γ is greater than one, then the diversification rate decreases through time. Model C assumes an abrupt change in rate before and after some breakpoint in the past. See McKenna & Farrell [98] for a visual comparison between these models. For model C, optimal time points for a shift in rate of diversification suggested by the LTT plot were tested. To detect and locate significant diversification rate shifts we used the Δ1, Δ2, and the Slowinski and Guyer (SG) [99] statistic (implemented in SymmeTREE 1.1 [100]), and the relative cladogenesis (RC) test (implemented in END-EPI).

### Analysis of ancestral areas

We estimated ancestral areas of Burmanniaceae with a dispersal-vicariance analysis using DIVA 1.1 [101]. Species distributions were scored using floristic regions as described by Takhtajan [102]. The ingroup taxa used in this study are distributed over 12 regions: Guineo-Congolian Region, Sudano-Zambezian Region, Madagascan Region, Indian Region, Indochinese Region, Malaysian Region, Caribbean Region, Amazonian Region (including Guyana Highlands), Brazilian Region, Eastern Asiatic Region, Northeast Australian Region, North American Atlantic Region. The number of unit areas allowed in ancestral distributions was restricted to two with the maxareas option in DIVA. To estimate the number of dispersals between the New and Old World the DIVA analysis was repeated with the terminals scored for presence in either the New World or the Old World.

## Abbreviations

18S rDNA: Nuclear small subunit ribosomal DNA; AIC: Akaike information criterion; CR test: Constant rate test; GTR: General time reversible model (a model of DNA sequence evolution); hLRT: Hierarchical likelihood ratio test; I + G: Invariant sites plus gamma distribution; ITS: Internal transcribed spacer; LTT plot: Lineage-through-time plot; MCMC: Markov chain Monte Carlo (a simulation method used to approximate the posterior probability of trees); *nad1 b-c*: The intron between the b and c exons of subunit one of the mitochondrial gene for NADH dehydrogenase; PL: Penalized likelihood; RC test: Relative cladogenesis test; SG statistic: Slowinski and Guyer statistic.

## Authors' contributions

VM conceived the study, gathered the molecular data, carried out the analyses, and wrote and edited the manuscript. LWC participated in the design of the study, helped with the analyses and writing of the manuscript. BL participated in the molecular study and the sequence alignment. MNS collected samples and helped drafting the manuscript. SH contributed to the data interpretations and writing of the manuscript. EFS participated in the coordination of the study and helped drafting the manuscript. All authors read and approved the final manuscript.

## Supplementary Material

Additional file 1 **GenBank accessions**. List of voucher numbers and Genbank accession numbers. Click here for file
