# Supplementary material for: Diversification of myco-heterotrophic angiosperms: Evidence from Burmanniaceae
Source: BMC Evol Biol. 2008 Jun 23;8:178. doi: 10.1186/1471-2148-8-178 (PMC2492876; doi:10.1186/1471-2148-8-178)
Supplement: Additional file 1 — GenBank accessions. List of voucher numbers and Genbank accession numbers. [file 1471-2148-8-178-S1.doc]

Appendix 1. Voucher information and GenBank accession numbers for Burmanniaceae taxa used in this study.

***Taxon***—GenBank accessions: 18S rDNA, *nad1 b-c,* ITS; Voucher; Locality. — = missing data.

***Apteria aphylla*** (Nutt.) Barnh. ex Small—DQ786035, DQ786093, EU816729; Chase 156, NCU; USA. ***Apteria aphylla*** (Nutt.) Barnh. ex Small—DQ786034, DQ786094, EU816730; Maas et al. 9662, U; Guyana. ***Burmannia sp.*** —EU816702, DQ887985, EU816735; Dessein 1021, BR; Zambia. ***Burmannia alba*** Mart.—DQ786074, DQ768133, EU816750; Nakajima et al. 323, U; Brazil. ***Burmannia bicolor*** Mart. —DQ786073, DQ786134, EU816752; Maas et al. 9649, U; French Guiana. ***Burmannia bicolor*** Mart.—DQ786072, DQ786132, EU816753; Ruysschaert 0636, GENT; Surinam. ***Burmannia biflora*** L.—DQ786070, DQ786142, EU816751; Chase 157, NCU; USA. ***Burmannia capitata*** (Walt. ex Gmel.) Mart.—DQ786066, DQ786128, EU816732; Neyland 958, MCN; USA. ***Burmannia capitata*** (Walt. ex Gmel.) Mart.—DQ786065, DQ786129, EU816733; Maas et al. 9606, U; French Guiana. ***Burmannia coelestis*** Don—DQ786068, DQ786123, EU816739; Cameron s.n., NCU; Malaysia. ***Burmannia congesta*** (Wright) Jonk.—DQ786061, DQ786120, EU816711; Jongkind 5463, WAG; Liberia. ***Burmannia congesta*** (Wright) Jonk.—EU420993, EU816757, EU816712; Merckx et al. 119, LV; Cameroon. ***Burmannia damazii*** Beauverd—DQ786071, DQ786127, EU816731; da Silva et al. 2195, U; Brazil. ***Burmannia disticha*** L.—U59947, DQ786124, EU816738; Wilkin 1017, K; Thailand. ***Burmannia flava*** Mart.—DQ786076, DQ786131, EU816755; Jansen-Jacobs et al. 5379, U; Guyana. ***Burmannia flava*** Mart.—DQ786077, DQ786130, EU816756; daSilva et al. 2087, U; Brazil. ***Burmannia hexaptera*** Schltr. —EU420994, EU816758, EU816734; Merckx 101, LV, Cameroon. ***Burmannia itoana*** Mak.—DQ786078, DQ786145, EU816740; Kun-Ping Lo 821, PPI; Taiwan. ***Burmannia juncea*** Sol. ex R.Br.—DQ786063, DQ786143, EU816737; Harwood 1499, BR; Australia. ***Burmannia latialata*** Pobég. —DQ786062, DQ786125, EU816746; Jongkind 5923, WAG; Gabon. ***Burmannia ledermannii*** Jonk.—DQ786079, DQ786135, EU816744; van Royen 4478, L; New Guinea. ***Burmannia longifolia*** Becc.—AF309398, DQ786138, EU816736; Cameron s.n., NCU; Malaysia. ***Burmannia lutescens*** Becc.—AF309401, DQ786144, EU816749; Caddick 352, K; Malaysia. ***Burmannia madagascariensis*** Mart.—AF309399, DQ786126, EU816742; Caddick 312, K; Madagascar. ***Burmannia madagascariensis*** Mart.—EU420995, EU816759, EU816743; De Block 1978, BR; Madagascar. ***Burmannia nepalensis*** (Miers) Hook.f.— EU816703, —, EU816748; Mueller 06-941, E; China. ***Burmannia oblonga*** Ridl.—DQ786064, DQ786140, EU816747; Wilkin 866, K; Thailand. ***Burmannia pusilla*** (Wall. ex Miers) Thw.—DQ786075, DQ786136, EU816745; Madhusoodanan s.n., U; India. ***Burmannia sphagnoides***Becc.—AF309400, DQ786137, —; Caddick 348, K; Malaysia. ***Burmannia stuebelii*** Hieron. and Schltr.—DQ786067, DQ786139, EU816754; Weigend 98/420, K; Peru. ***Burmannia wallichii*** (Miers) Hook.f.—DQ786069, DQ786141, EU816741; Zhang s.n., K; Hong Kong. ***Campylosiphon purpurascens*** Benth. —EU420996, EU816760, EU816710; Banki 1257, U; Guyana. ***Cymbocarpa refracta*** Miers—DQ786038, DQ786095, EU816725; Kress s.n., US; Costa Rica. ***Cymbocapra saccata*** Sandw.—DQ786039, EU816726; Maas and Westra 4242, U; Guyana. ***Dictyostega orobanchoides*** (Hook.) Miers—DQ786056, DQ786119, EU816713; Maas et al. 9620, U; French Guiana. ***Gymnosiphon aphyllus*** Bl.—AF309402, DQ786102, EU816721; Caddick 353, K; Malaysia. ***Gymnosiphon bekensis*** Letouzey—EU420998, EU816761, EU816724; Merckx et al. 117, LV; Cameroon. ***Gymnosiphon breviflorus*** Gleason—DQ786036, DQ786101, EU816717; Maas et al. 9660, U; Guyana. ***Gymnosiphon breviflorus*** Gleason—DQ786040, DQ786099, EU816718; Maas et al. 9676, U; Guyana. ***Gymnosiphon breviflorus*** Gleason—DQ786037, DQ786100, EU816719; Maas et al. 9675, U; Guyana. ***Gymnosiphon capitatus*** (Benth.) Urb.—DQ786054, DQ786114, EU816715; Maas et al. 9616, U; Guyana. ***Gymnosiphon divaricatus***(Benth.) Benth. and Hook.— DQ786042, DQ786105, EU816727; Fuchs and Zanella 21810, U; Colombia. ***Gymnosiphon divaricatus*** (Benth.) Benth. and Hook.—DQ786044, DQ786107, EU816728; Maas et al. 9657, U; Guyana. ***Gymnosiphon longistylus*** (Benth.) Hutch. and Dalziel—DQ786051, DQ786103, EU816723; Breteler et al. 9705, WAG; Gabon. ***Gymnosiphon minutus*** Snelders and Maas—DQ786047, DQ786108, EU816722; Maas et al. 9668, U; Guyana. ***Gymnosiphon panamensis***Jonk.—DQ786055, DQ786115, —; Wendt et al. 2312, U; Mexico. ***Gymnosiphon recurvatus*** Snelders and Maas—DQ786050, DQ786116, EU816716; Maas and Westra 4153, U; Guyana. ***Gymnosiphon suaveolens***(Karst.) Urb.—U59942, DQ786097, —; Nickrent 3005, SIU; Costa Rica. ***Gymnosiphon usambaricus***Engl.—DQ786053, DQ786113, EU816720; Bytebier 1217, BR; Kenya. ***Hexapterella gentianoides***Urb.—DQ786057, DQ786118, EU816714; Maas et al. 9614, U; French Guiana. ***Pandanus tectorius*** Parkinson ex Du Roi (outgroup)—AY952391, EU816762, EU816709; Merckx 201, LV.

Appendix 2. GenBank accessions of 18S rDNA sequences used in the age estimation analyses, excluding all 18S rDNA sequences listed in Appendix 1.

**Outgroups:** *Amborella trichopoda* (U42497) **Acorales:** *Acorus calamus* (L24078), *Acorus gramineus* (AF197584) **Alismatales:** *Alisma plantago-aquatica* (AF197585), *Pistia stratiotes* (AF168869), *Potamogeton berchtoldii* (DQ007410), *Scheuchzeria palustris* (AF069202), *Zostera noltii* (AF207058), *Tofieldia calyculata* (AF207043), *Pleea tenuifolia* (AF206995), *Orontium aquaticum* (AF293753), *Spathiphyllum wallisii* (AF207023); *Ranalisma rostratum* (AY952388), *Aponogeton crispus* (AF168826), *Calla palustris* (AF168829), *Gymnostachys anceps* (AF069200), *Halodule uninervis* (AY952401), *Syringodium filiforme* (AF168876), *Halophila ovalis* (AY952400), *Hydrilla verticillata* (AY952399), *Hydrocharis dubia* (AY952398), *Ottelia acuminata* (AY952392), *Thalassia hemprichii* (AY952386), *Vallisneria asiatica* (AY952384), *Triglochin maritimum* (AF197589), *Lilaea scilloides* (AF168857), *Hydrocleys nymphoides* (AY952397), *Limnocharis flava* (AY952395), *Posidonia oceanica* (AY491942), *Potamogeton perfoliatus* (AY952389), *Zannichellia palustris* (AF168883), *Blyxa japonica* (AY952406), *Caldesia oligococca* (AY942405), *Enhalus acoroides* (AY952403) **Petrosaviales:** *Petrosavia stellaris* (AF206987), *Japanolirion osense* (AF206942) **Pandanales:** *Cyclanthus bipartitus* (AF168837), *Pandanus tectorius* (AY952391), *Stemona japonica* (AF207028), *Sciaphila albescens* (EU816705), *Sciaphilla densiflora* (EU816704), *Kupea martinetugei* (EU816706), *Barbacenia elegans* (AF206861), *Carludovica palmata* (AF293756), *Sphaeradenia pendula* (AF207024), *Freycinetia scandens* (AF206915), *Croomia japonica* (AF309408), *Pentastemona sumatrana* (AF309406), *Stichoneuron caudatum* (AF168875), *Croomia pauciflora* (AF168835) **Dioscoreales:** *Dioscorea althaeoides* (EU420997), *Dioscorea bulbifera* (AF069203), *Dioscorea polygonoides* (AF206903), *Dioscorea prazeri* (DQ786089), *Dioscorea rockii* (DQ786090), *Stenomeris dioscoreifolia* (DQ786087), *Trichopus sempervirens* (AF309395), *Trichopus zeylanicus* (AF309394), *Tacca artocarpifolia* (AF309397), *Tacca chantrieri* (DQ786086), *Tacca integrifolia* (DQ786085), *Tacca leontopetaloides* (EU420999), *Tacca palmata* (EU421000), *Tacca palmatifida* (DQ786084), *Tacca plantaginea* (U42063), *Afrothismia hydra* (DQ786083), *Haplothismia exannulata* (DQ786082), *Thismia aseroe* (AF309404), *Thismia clavigera* (AF309405), *Thismia panamensis* (DQ786081), *Thismia rodwayi* (AF309403), *Thismia taiwanensis* (DQ786080), *Lophiola americana* (EU186218), *Lophiola aurea* (DQ786091), *Metanarthecium luteo-viride* (AF309410), *Narthecium ossifragum* (AF309411), *Nietneria paniculata* (EU186219) **Liliales:** *Bomarea hirtella* (AF206871), *Colchicum autumnale* (U42072), *Luzuriaga latifolia* (AF233091), *Lilium superbum* (AF206952), *Trillium erectum* (AF207048), *Smilax glauca* (AF207022) **Asparagales:** *Agave ghiesbreghtii* (AF206841), *Allium thunbergii* (AF168825), *Asparagus falcatus* (AF069205), *Borya septentrionalis* (AF206872), *Ixiolirion tataricum* (AF206940), *Aristea glauca* (AF206854), *Geosiris sp.* (EU816707), *Gladiolus buckerveldii* (L54062), *Iris japonica* (AY952396), *Isophysis tasmanica* (L54063), *Aplectrum hyemale* (U59937), *Apostasia stylidioides* (AF135207), *Cleistes divaricata* (AF135207), *Corallorhiza maculata* (U59940), *Cymbidium kanran* (CKA27124), *Cypripedium calceolus* (AF069208), *Cyrtosia septentrionalis* (AF135198), *Dendrobium nobile* (AB027309), *Diuris sulphurea* (AF135196), *Eburophyton austinae* (U59949), *Epistephium subrepens* (AF135200), *Eriaxis rigida* (AF135201), *Erythrochis cassythoides* (AF135199), *Isotria verticillata* (AF135204), *Lecanorchis multiflora* (AF135203), *Neottia nidus-avis* (U59948), *Neuwedia veratrifolia* (AF135208), *Oncidium excavatum* (OEU42791), *Oncidium ornithoglossum* (AF168864), *Oncidium sphacelatum* (U59939), *Orchis quadripunctata* (AF135206), *Paphiopedilum coccineum* (AJ604527), *Paphiopedilum delenatii* (AF135206), *Paphiopedilum helenae* (AJ293100), *Paphiopedilum micranthum* (AJ303203), *Paphiopedilum sp.* (AF168868), *Rhizanthella gardneri* (AF135197), *Spiranthes cernua* (AF135195), *Triphora trianthophora* (AF135193), *Tropidia sp.* (AF135192), *Vanilla aphylla* (AF135202), *Wullschlaegelia calcarata* (EU816708), *Tecophilaea cyanocrocus* (AF207036), *Xeronema callistemon* (AF207056) **Arecales:** *Areca triandra* (AY952409), *Trachycarpus wagnerianus* (D29773) **Commelinales:** *Tradescantia ohiensis* (AF069213), *Anigozanthos flavidus* (AF069214), *Hanguana malayana* (AF387604), *Philydrum lanuginosum* (AY952390), *Pontederia cordata* (AF206998) **Zingiberales:** *Canna indica* (AF069221), *Costus barbatus* (AF069222), *Maranta bicolor* (U42079), *Musa acuminata* (AF069226), *Zingiber officinale* (AB047730) **Dasypogonaceae:** *Dasypogon bromeliifolius* (AJ417898) **Poales:** *Ananas comosus* (D29786), *Cyperus albostriatus* (AF168838), *Juncus effusus* (AF206944), *Elegia sp.* (AF069219), *Eriocaulon septangulare* (AY952402), *Flagellaria indica* (AF206913), *Joinvillea ascendens* (AF168855); *Lolium multiflorum* (AY846367), *Mayaca aubletii* (AF168859), *Restio tetraphyllus* (AF207006), *Sparganium eurycarpum* (AF069220), *Typha latifolia* (AF168880), *Xyris difformis* (AF16881).
